# Gamma Knife Radiosurgery-Based Combination Treatment Strategies Improve Survival in Patients With Central Nervous System Metastases From Epithelial Ovarian Cancer: A Retrospective Analysis of Two Academic Institutions in Korea and Taiwan

**DOI:** 10.3389/fonc.2021.719936

**Published:** 2021-08-27

**Authors:** Yen-Ling Lai, Jun-Hyeok Kang, Che-Yu Hsu, Jung-Il Lee, Wen-Fang Cheng, Yu-Li Chen, Yoo-Young Lee

**Affiliations:** ^1^Department of Obstetrics and Gynecology, Hsin-Chu Branch, National Taiwan University Hospital, Hsin−Chu, Taiwan; ^2^Department of Obstetrics and Gynecology, College of Medicine, National Taiwan University, Taipei, Taiwan; ^3^Department of Obstetrics and Gynecology, Uijeongbu Eulji Medical Center, Eulji University School of Medicine, Uijeongbu-si, South Korea; ^4^Division of Radiation oncology, Department of Oncology, National Taiwan University Hospital, Taipei, Taiwan; ^5^Department of Neurosurgery, Samsung Medical Center, Sungkyunkwan University School of Medicine, Seoul, South Korea; ^6^Division of Gynecologic oncology, Department of Obstetrics and Gynecology, Samsung Medical Center, Sungkyunkwan University School of Medicine, Seoul, South Korea

**Keywords:** ovarian cancer, CNS metastasis, brain metastasis, whole brain radiation therapy, gamma knife radiosurgery

## Abstract

Central nervous system (CNS) metastases from epithelial ovarian cancer (EOC) are rare. We investigated the clinico-pathological prognostic factors of patients with CNS metastases from EOC and compared the outcomes of various treatment modalities. We retrospectively reviewed the records of patients with CNS metastases from EOC between 2000 and 2020. Information on the clinical and pathological characteristics, treatment, and outcomes of these patients was retrieved from Samsung Medical Center and National Taiwan University Hospital. A total of 94 patients with CNS metastases were identified among 6,300 cases of EOC, resulting in an incidence of 1.49%. Serous histological type [hazard ratio (HR): 0.49 (95% confidence interval [CI] 0.25-0.95), *p*=0.03], progressive disease [HR: 2.29 (95% CI 1.16-4.54), *p*=0.01], CNS involvement in first disease relapse [HR: 0.36 (95% CI 0.18-0.70), *p*=0.002], and gamma knife radiosurgery (GKS)-based combination treatment for EOC patients with CNS lesions [HR: 0.59 (95% CI 0.44-0.79), *p*<0.001] significantly impacted survival after diagnosis of CNS metastases. In a subgroup analysis, superior survival was observed in patients with CNS involvement not in first tumor recurrence who underwent GKS-based combination therapeutic regimens. The survival benefit of GKS-based treatment was not significant in patients with CNS involvement in first disease relapse, but a trend for longer survival was still observed. In conclusion, GKS-based combination treatment can be considered for the treatment of EOC patients with CNS metastases. The patients with CNS involvement not in first disease relapse could significantly benefit from GKS-based combination strategies.

## Introduction

Epithelial ovarian cancer (EOC) is the leading cause of mortality among female genital cancers (1). Approximately 70%-80% of patients with EOC will relapse despite optimal debulking surgery and effective adjuvant chemotherapy (2, 3). Recurrence usually presents in the pelvis and abdomen, with peritoneal or lymphatic spread (4). Brain metastasis as a consequence of hematogenous dissemination is very rare, with an incidence of only 1-2% in patients with EOC (5–7).

Patients with intraabdominal recurrence of EOC may respond well to chemotherapy. However, the efficacy of chemotherapy is insufficient in treating brain metastases because it has limitations in penetrating the blood-brain barrier. Surgical resection, whole brain radiation therapy (WBRT), and stereotactic radiosurgery are common treatment modalities for brain metastases. Despite these therapeutic options, there are no established guidelines for the management of brain metastasis in EOC patients and the prognosis of these cases remains poor (5, 8).

For decades, WBRT has been the standard treatment for brain metastases. Recently, stereotactic radiosurgery has become a feasible option for the treatment of brain metastases because of its short treatment course, high local efficacy, and avoidance of impairing cognitive function (9, 10). Numerous studies have investigated the impact of stereotactic radiosurgery on brain metastases from different cancer types (10). Only some small series reported the results of gamma knife radiosurgery (GKS, a kind of stereotactic radiosurgery) for the treatment of cerebral metastases due to the rarity of brain metastases from EOC (11, 12).

In this first collaborative study from two large academic institutions in Korea and Taiwan, we analyzed clinico-pathological prognostic factors to predict outcomes in EOC patients with metastases of the central nervous system (CNS), including the brain, spinal cord, and both. The potential therapeutic strategies for these cases were also investigated.

## Patients and Methods

Patients who had CNS metastases (brain, spinal cord, or both) from EOC between January 2000 and December 2020 at Samsung Medical Center (SMC), Seoul, Korea, and National Taiwan University Hospital (NTUH), Taipei, Taiwan, were retrospectively reviewed. Among patients with spinal cord metastases, only patients with high cervical involvement (C1-C2 level) amenable to GKS treatment were included in this study. This study was conducted in accordance with ethical principles and approved by the institutional review boards of SMC and NTUH (ethical approval code: 2021-02-126-001 and HCH 110-07-049). Informed consent was exempted by approval of the ethics committee.

We extracted the demographic and clinical characteristics of the studied population from medical charts. These parameters included age at initial diagnosis, International Federation of Gynecology and Obstetrics (FIGO) stage of disease (13), histological subtype, tumor grading, pre-treatment serum levels of cancer antigen 125 (CA-125), primary treatment modalities for EOC, types of cytoreductive surgeries, lymph node status (i.e., metastasis), response to treatment of primary disease, progression-free interval (PFI), and CNS involvement in first disease recurrence or not. The number and location of CNS metastases, symptoms and signs of CNS metastases, interval between treatment of primary disease and CNS metastases, treatment modalities for patients with CNS metastases, response to treatment of CNS metastatic tumors, CNS recurrence-free interval, and location and treatment of second CNS relapse were also reviewed.

Primary treatment modalities for EOCs included primary debulking surgeries followed by platinum-based adjuvant chemotherapy; platinum-based neoadjuvant chemotherapy followed by interval debulking surgeries, and then platinum-based adjuvant chemotherapy; platinum-based chemotherapy alone; and surgery alone. The type of cytoreductive surgery was based on the largest diameter of the postoperative residual tumor. The optimal debulking surgeries were defined as residual implants <1 cm, and the suboptimal surgeries were residual tumor ≥1 cm. The response to treatment of primary disease was defined according to Response Evaluation Criteria In Solid Tumors (RECIST) version 1.1. Non-progressive disease included complete response, partial response, and stable disease. The PFI was considered as the time from the date of completion of treatment of primary disease to that of first disease relapse. The interval between treatment of primary disease and CNS metastases was defined as the time from the date of completing treatment of primary disease to that of CNS metastases.

**Figure 1:**
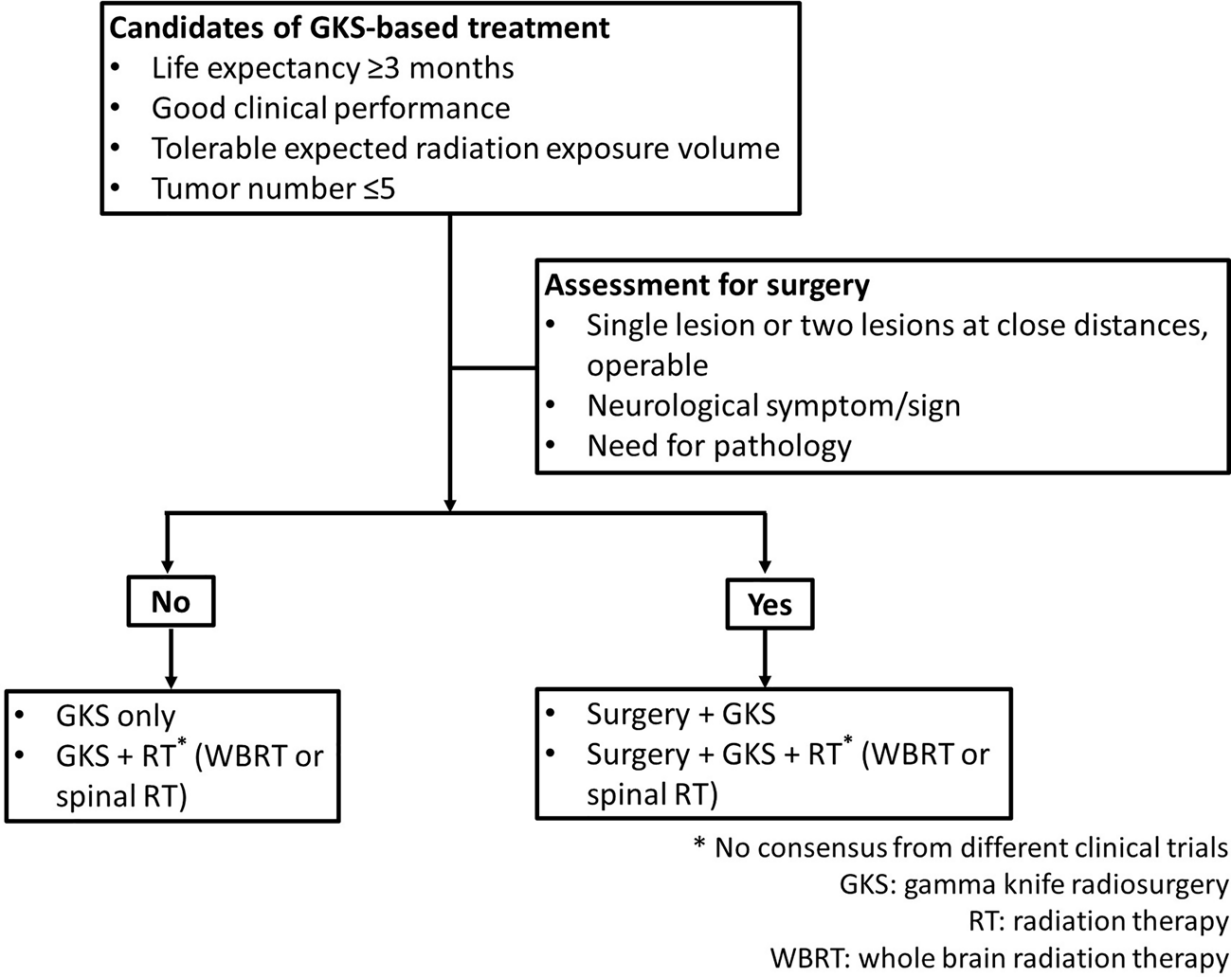
Assessment for GKS-based treatment strategies in clinical practice.

Therapeutic modalities for patients with CNS metastases included RT, GKS, surgical resection, chemotherapy, and combination strategies. GKS was applied to brain metastases and high cervical lesions. In this study, RT included WBRT and spinal RT. At present, there is no established treatment guideline for CNS metastasis from ovarian cancers because of rarity of such patients. For these cases, gynecologic oncologists at the two institutions worked with a multidisciplinary team to determine the treatment options. In general, patients with life expectancy ≥3 months, good clinical performance, tolerable expected cumulative volume of radiation, and the number of target lesions ≤5 (defined as oligometastatic state in this study) would receive GKS only, GKS and RT (WBRT or spinal RT), or surgery plus GKS and/or RT (WBRT or spinal RT). Patients with life expectancy <3 months, the number of target lesions were >5, or poor clinical condition for surgery were treated with RT (WBRT or spinal RT) only. Regarding extra-CNS metastatic tumors, there were rarely oligometastases in ovarian cancer. The spread pattern of ovarian cancer is usually disseminated in abdominal and pelvic cavity. Chemotherapy was used to control extra CNS lesions. Requirements for candidates treated with GKS-based treatment were presented in **Figure 1**. In this study, GKS-based treatment included GKS only and GKS-based combination treatment groups.

GKS-based combination treatment meant GKS combined with other treatment modalities which included WBRT, spinal RT, chemotherapy, or surgery. In addition to GKS, patients in GKS-based combination treatment group (i.e. GKS + other modalities group) received at least one of other treatment modalities. Therefore, cases in GKS-based combination treatment group included those receiving GKS and chemotherapy, GKS and RT (WBRT or spinal RT) with/without chemotherapy, surgery plus GKS with/without chemotherapy, or surgery plus GKS and RT (WBRT or spinal RT) with/without chemotherapy. Because of the small number in respective group, these cases were classified into one category, GKS-based combination treatment group for analysis.

Treatment planning including dose prescription was performed by a neurosurgeon. Prescription dose to the tumor margin was selected based on the multiple factors including tumor volume, location and number of the lesions, beam-on time, duration of treatment, patient’s clinical condition, and previous history of radiation exposure. Generally, dose selection for GKS was determined according to the RTOG protocol 90-05 (13). Recently, repeated GKS has been recommended if the volume of lesion is approximately over 15 cm^3^. If brain lesion is surgically removed, GKS can also be recommended in the tumor resection site within 2 weeks. Vice versa, if the patient receives the GKS upfront, the brain lesion can also be surgically removed within 1 week. With clinical improvement of neurologic disturbances by GKS treatment, systemic chemotherapy for extra CNS tumors can be considered.

After completing the treatment of primary disease, regular surveillance of the EOC patients was arranged every 3 months for 3 years, and then every 6 months thereafter. Computed tomography (CT) or magnetic resonance imaging (MRI) was performed for suspected disease recurrence. Regarding CNS surveillance strategy, first brain MRI was performed 2-3 months after the completion of GKS and in 3-month intervals to evaluate responses. Depending on a patient’s clinical condition and response to treatment for CNS lesions, GKS can be repeated every 3-6 months. If any neurological symptoms develop, immediate assessments were needed. Recurrence was defined according to the Gynecological Cancer Intergroup (GCIG) criteria for CA-125 progression, RECIST 1.1 for image studies, or biopsy-proven disease. RECIST 1.1 criteria was also used to evaluate patients’ responses to therapies for their CNS metastatic lesions. Survival from CNS metastasis was calculated as the time from the date of diagnosis of CNS metastases to death or last follow-up. CNS recurrence-free interval was defined as the time from the completion of treatment of CNS metastatic tumors to local relapse or last follow-up.

All results are presented as frequency and rate for categorical variables or median and range for continuous variables. A Cox proportional hazards regression model was used for univariable and multivariable analyses to assess the independence of different prognostic factors. The Kaplan-Meier method was used to calculate survival. The log-rank test was used to compare survival between groups. *P*<0.05 was considered significant. Statistical analyses were performed using SPSS software (IBM SPSS Statistics for Windows, version 25.0) and MedCalc software 14.12.0 (MedCalc Software bvba, Belgium).

## Results

### Characteristics of the Studied Population

In the study period, 6,300 consecutive patients were diagnosed with EOC at SMC (n=3,871) and NTUH (n=2,429). As shown in **Table 1**, a total of 94 women who met inclusion criteria were identified, resulting in an incidence of 1.49%. More patients were identified between 2010 and 2020 than the first half of the screened time period. However, the incidence of 1.31% between 2000 and 2009 (1.81% at NTUH and 0.98% at SMC) was similar to the 1.61% between 2010 and 2020 (1.68% at NTUH and 1.56% at SMC). The basic characteristics of EOC patients with CNS metastases from NTUH and SMC were also presented in **Table 1**.

**Table 1:** Basic characteristics of this studied population from SMC and NTUH.

	SMC (N = 52)	NTUH (N = 42)	Total (N = 94)
**Age (median)**	55	56	56.6
<50 years	15 (28.8%)	15 (35.7%)	30 (31.9%)
≧50 years	37 (71.2%)	27 (64.3%)	64 (68.1%)
**Year of diagnosis**			
2000-2009	14 (26.9%)	17 (40.5%)	31 (33.0%)
2010-2020	38 (73.1%)	25 (59.5%)	63 (67.0%)
**Histology**			
Serous type	43 (82.7%)	31 (73.8%)	74 (78.7%)
Non-serous type	9 (17.3%)	11 (26.2%)	20 (21.3%)
Endometrioid type	1 (1.9%)	2 (4.8%)	3 (3.2%)
Clear cell type	2 (3.8%)	4 (9.5%)	6 (6.4%)
Mucinous type	1 (1.9%)	2 (4.8%)	3 (3.2%)
Others	5 (9.7%)	3 (7.1%)	8 (8.5%)
Grade^a^			
Grade 1	2 (3.8%)	0 (0.0%)	2 (2.1%)
Grade 2	5 (9.7%)	3 (7.1%)	8 (8.5%)
Grade 3	42 (80.8%)	36 (85.7%)	78 (83%)
**FIGO stage**			
Stage I & II	4 (7.7%)	7 (16.7%)	11 (11.7%)
Stage III & IV	48 (92.3%)	35 (83.3%)	83 (88.3%)
Pre-treatment CA-125, U/ml (median)^a^	985	1166.7	1005.5
**Treatment of primary disease**			
Surgery + adjuvant CT	45 (86.6%)	31 (73.8%)	76 (80.9%)
NACT + surgery + adjuvant CT	6 (11.5%)	7 (16.7%)	13 (13.8%)
Surgery only	1 (1.9%)	1 (2.4%)	2 (2.1%)
CT only	0 (0.0%)	3 (7.1%)	3 (3.2%)
Surgery type^a^			
Optimal debulking surgery	39 (75.0%)	24 (57.1%)	63 (67.0%)
Suboptimal debulking surgery	12 (23.1%)	16 (38.0%)	28 (29.8%)
LN involvement^a^			
No LN involvement	13 (25.0%)	9 (21.4%)	22 (23.4%)
LN involvement	32 (61.5%)	13 (31.0%)	45 (47.9%)
Response to treatment of primary disease^a^			
Complete response	34 (65.4%)	28 (66.7%)	62 (66.0%)
Non-complete response	15 (28.8%)	15 (35.7%)	30 (31.9%)
Partial response	7 (13.5%)	4 (9.5%)	11 (11.7%)
Stable disease	6 (11.5%)	1 (2.4%)	7 (7.4%)
Progressive disease	2 (3.8%)	10 (23.8%)	12 (12.8%)
**Progression-free interval**			
<6 months	2 (3.8%)	11 (26.2%)	13 (13.8%)
6-12 months	25 (48.1%)	11 (26.2%)	36 (38.3%)
>12 months	25 (48.1%)	20 (47.6%)	45 (47.9%)
**CNS involvement in first disease relapse**			
Yes	19 (36.5%)	10 (23.8%)	29 (30.9%)
No	33 (63.5%)	32 (76.2%)	65 (69.1%)

N, number; SMC, Samsung Medical Center; NTUH, National Taiwan University Hospital; FIGO, International Federation of Gynecology and Obstetrics; CA-125, cancer antigen 125; +, and; NACT, neoadjuvant chemotherapy; CT, chemotherapy; LN, lymph node.

a Some patients have unavailable data.

For these cases, the median duration of follow-up from initial EOC diagnosis until the date of death or last visit was 44.4 months (range, 4.6-165.6 months). In the studied population (**Table 1**), the median age at diagnosis of EOC was 56.0 years (range, 31.0-77.0 years). Seventy-four (78.7%) cases had serous adenocarcinoma, 78 (83.0%) had grade 3 tumors, and 83 (88.3%) had advanced disease. The median pre-treatment serum CA-125 level was 1,005.5 U/mL (range, 5.0-39,370.0). Seventy-six (80.9%) patients underwent primary debulking surgeries followed by adjuvant chemotherapy. Sixty-three (67.0%) women had a <1 cm residual tumor after cytoreductive surgery. Sixty-two (66.0%) cases were classified as a complete response and 45 (47.9%) had PFI >12 months after treatment of primary disease. At the time of first tumor relapse, 29 (30.9%) patients had CNS involvement. The remaining 65 (69.1%) CNS metastases were not detected in the first disease recurrence.

As presented in **Table 2**, most EOC patients with CNS metastases had lesions confined to the brain, experienced multiple CNS involvement with lesion number ≤5, and underwent one or more neurological disturbances. The majority of patients (27/29, 93.1%) developed CNS metastases at first disease relapse ≤24 months after completing treatment of EOC. Among those cases with CNS involvement not in the first tumor recurrence, 40 of 65 (61.5%) were diagnosed >24 months after completing EOC treatment. In the studied population, 90 (95.7%) patients underwent radiation-based strategies to control the tumors. Among these cases, 16 (17.0%) were treated with RT alone and 25 (26.6%) with GKS alone. Twenty-nine women underwent RT with surgical resection or chemotherapy and 20 (21.3%) underwent GKS combined with RT (WBRT or spinal RT), surgical resection, or chemotherapy. Among 6 patients with spinal cord metastases, 2 women had isolated high cervical lesions. One was treated with GKS alone, and the other underwent GKS followed by fractionated spinal RT. The remaining four patients had multiple spinal cord metastases, including high cervical involvement and all received fractionated spinal RT.

**Table 2:** Clinical characteristics of ovarian cancer patients with CNS metastases (94 cases).

	CNS involvement in first disease relapse (N = 29)	CNS involvement not in first disease relapse (N = 65)
**CNS location**		
Brain	27 (93.1%)	58 (89.2%)
Spinal cord^a^	0 (0.0%)	6 (9.2%)
Both brain and spinal cord	2 (6.9%)	1 (1.5%)
**Number of CNS lesions**		
<3	14 (48.3%)	30 (46.2%)
3-5	14 (48.3%)	31 (47.7%)
>5	1 (3.4%)	4 (6.2%)
**Symptoms/signs** **^b^**		
Headache	16 (55.2%)	29 (44.6%)
Ataxia	5 (17.2%)	7 (10.8%)
Altered mental status	1 (3.4%)	12 (18.5%)
Dizziness	10 (34.5%)	19 (29.2%)
Seizures	1 (3.4%)	6 (9.2%)
Nausea/vomiting	9 (31.0%)	13 (20.0%)
Limb weakness	10 (34.5%)	24 (36.9%)
Vision changes	1 (3.4%)	5 (7.7%)
Others^c^	1 (3.4%)	7 (10.8%)
No obvious symptom	0 (0.0%)	3 (4.6%)
**Interval between treatment of primary disease and CNS relapse**		
<12 months	14 (48.3%)	7 (10.8%)
12-24 months	13 (44.8%)	18 (27.7%)
>24 months	2 (6.9%)	40 (61.5%)
**Treatment of CNS relapse**		
RT^d^ only	4 (13.8%)	12 (18.5%)
RT^d^ + other modalities^e^	9 (31.0%)	20 (30.8%)
GKS only	8 (27.6%)	17 (26.2%)
GKS + other modalities^f^	8 (27.6%)	12 (18.5%)
Surgery only	0 (0.0%)	1 (1.5%)
Supportive care	0 (0.0%)	3 (4.6%)

CNS, central nervous system; +, and; RT, radiation therapy; GKS, gamma knife radiosurgery.

a All these patients had spinal lesions at high cervical region. Two had isolated high cervical lesions, and the remaining four had multiple spinal cord lesions, including high cervical involvement.

b Some patients complained of multiple clinical manifestations.

c Others included paresthesia, paraplegia, back pain, incontinence and aphasia.

d RT included whole brain radiation therapy (WBRT) and spinal RT.

e Other modalities included chemotherapy, or surgery.

f Other modalities included RT (WBRT or spinal RT), chemotherapy, or surgery.

### Prognostic Factors Affecting Survival From CNS Metastases

Next, we investigated the prognostic factors influencing the survival of EOC patients with CNS metastases. The results of the univariable and multivariable Cox regression analyses are shown in **Table 3**. In the univariable analysis, tumor histology (*p*=0.002), response to primary treatment for EOC (*p*<0.001), CNS involvement in first disease relapse (*p*<0.001), and treatment modalities for patients with CNS metastases (*p*<0.001) were significantly associated with survival after diagnosis of CNS metastases (**Table 3**).

**Table 3:** Univariable and multivariable analysis of prognostic factors affecting survival from CNS metastasis.

	Univariable		Multivariable	
	HR (95% CI)^a^	*p* value	HR (95% CI)^a^	*p* value
**Age**				
[≧50 years / <50 year]	1.00 (0.60-1.67)	0.997		
**Histology**				
[serous / non-serous]	0.31 (0.15-0.63)	0.002	0.49 (0.25-0.95)	0.03
**Grade**				
[grade 3 / grade 1&2]	1.00 (0.48-2.11)	0.993		
**FIGO stage**				
[stage III&IV / stage I&II]	0.73 (0.33-1.61)	0.368		
**Treatment of primary disease**				
[NACT + surgery + adjuvant CT /surgery + adjuvant CT /surgery only / chemotherapy only]	1.01 (0.70-1.46)	0.954		
**Surgery type**				
[optimal / suboptimal]	1.02 (0.59-1.74)	0.956		
**LN involvement**				
[yes / no]	0.84 (0.45-1.58)	0.578		
**Response to treatment of primary disease**				
[PD / Non-PD]	2.77 (1.10-6.94)	<0.001	2.29 (1.16-4.54)	0.01
**Progression-free interval**				
[<6 months / 6-12 months / >12 months]	0.73 (0.50-1.06)	0.096		
**CNS involvement in first disease relapse**				
[yes / no]	0.33 (0.20-0.56)	<0.001	0.36 (0.18-0.70)	0.002
**Number of CNS lesions**				
[<3 / 3-5 / >5]	1.18 (0.34-4.11)	0.928		
**Interval between treatment of primary disease and CNS relapse**				
[<12 months / 12-24 months / >24 months]	1.09 (0.59-2.04)	0.099		
**Treatment of CNS relapse**				
[GKS + other modalities^b^ / GKS / RT^c^ + other modalities^d^ / RT^c^ only]	0.63 (0.48-0.81)	<0.001	0.59 (0.44-0.79)	<0.001

HR, hazard ratio; CI, confidence interval; CNS, central nervous system; FIGO, International Federation of Gynecology and Obstetrics; +, and; CT, chemotherapy; NACT, neoadjuvant chemotherapy; LN, lymph node; PD, progressive disease; GKS, gamma knife radiosurgery; RT, radiation therapy.

a Cox regression model.

b Other modalities included WBRT, chemotherapy or surgery.

c RT included whole brain radiation therapy (WBRT) and spinal RT.

d Other modalities included chemotherapy, or surgery.

**Figure 2:**
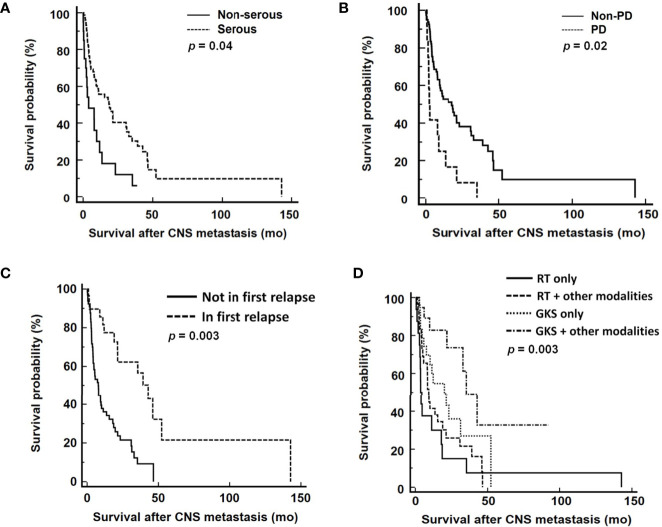
Survival analyses from diagnosis of CNS metastases according to **(A)** tumor histology, **(B)** response to primary treatment, **(C)** CNS involvement in first disease relapse, and **(D)** different treatment modalities. CNS, central nervous system; PD, progressive disease; RT, radiation therapy; GKS, gamma knife radiosurgery. RT included WBRT and spinal RT.

In the multivariable analysis, serous histological type [hazard ratio (HR): 0.49 (95% confidence interval (CI) 0.25-0.95), *p*=0.03], progressive disease [HR: 2.29 (95% CI 1.16-4.54), *p*=0.01], CNS involvement in first disease relapse [HR: 0.36 (95% CI 0.18-0.70), *p*=0.002], and GKS-based combination treatment for EOC patients with CNS lesions [HR: 0.59 (95% CI 0.44-0.79), *p*<0.001] significantly impacted survival after diagnosis of CNS metastases (**Table 3**). The median overall survival from diagnosis of CNS metastases was 11.9 months. Patients with serous carcinoma (*p*=0.04, **Figure 2A**), CNS involvement in first disease relapse (*p*=0.003, **Figure 2C**), or GKS-based combination treatment (*p*=0.003, **Figure 2D**) had better survival. However, survivals of patients in RT only, RT-based combination treatment, and GKS only groups did not show significant difference (*p*=0.14). Cases with tumor progression after primary treatment for EOC had worse survival (*p*=0.02, **Figure 2B**).

### Analyses of Local Control of CNS Metastases by Different Treatment Modalities

**Figure 3:**
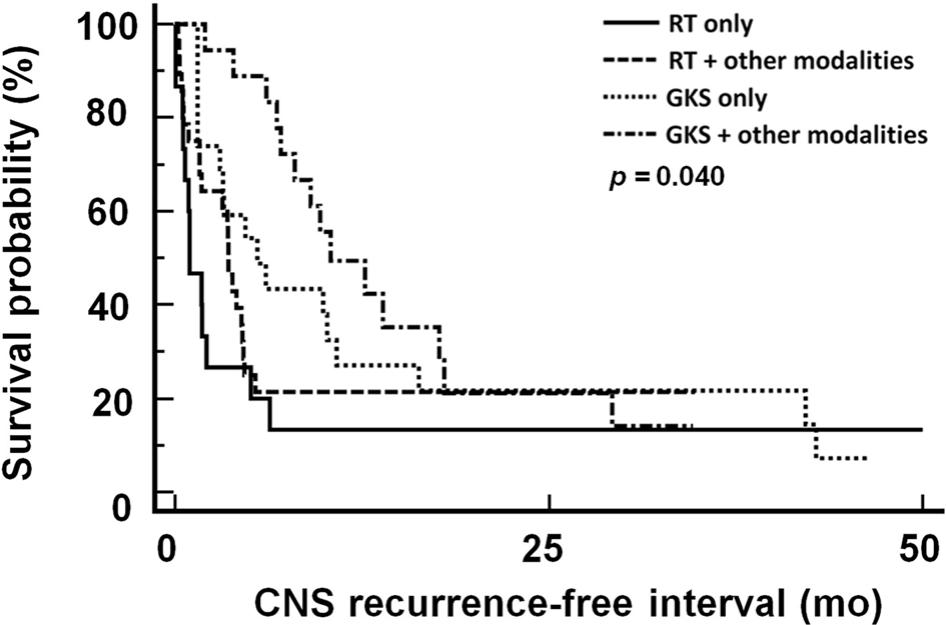
CNS recurrence-free interval in patients treated with different treatment modalities. CNS, central nervous system; RT, radiation therapy; GKS, gamma knife radiosurgery. RT included WBRT and spinal RT.

Therapeutic response rates of different treatment modalities were 43.75% in RT only group, 65.5% in RT-based combination group, 68.0% in GKS only group, and 80.0% in GKS-based combination group (**Table 4**). The median CNS recurrence-free interval was 4.3 month. As shown in **Figure 3**, patients treated with GKS-based combination strategies had longest CNS recurrence-free interval (*p*=0.04) in this studied population. Median CNS recurrence-free intervals of different treatment modalities were 1.0 month in RT only group, 3.6 months in RT-based combination group, 4.0 months in GKS only group, and 9.7 months in GKS-based combination group.

**Table 4:** Responses rates of treatment of CNS metastases.

	RT^a^ only (N = 16)	RT^a^ + other modalities^b^ (N = 29)	GKS only (N = 25)	GKS + other modalities^c^ (N = 20)
**Complete response**	2 (12.5%)	6 (20.7%)	5 (20%)	7 (35%)
**Partial response**	4 (25%)	10 (34.5%)	9 (36%)	8 (40%)
**Stable disease**	1 (6.25%)	3 (10.3%)	3 (12%)	1 (5%)
**Progressive disease**	8 (50%)	10 (34.5%)	7 (28%)	4 (20%)
**Not available**	1 (6.25%)	0 (0%)	1 (4%)	0 (0%)

CNS, central nervous system; +, and; RT, radiation therapy; GKS, gamma knife radiosurgery.

a RT included whole brain radiation therapy (WBRT) and spinal RT.

b Other modalities included chemotherapy, or surgery.

c Other modalities included RT, chemotherapy, or surgery.

Five cases in GKS-based combination group, 5 in GKS only group, and 7 in RT-based combination group did not have CNS recurrence after treatment (**Table 5**). Among these patients with recurrent CNS tumors, 10 in GKS-based combination group, 19 in GKS only group, 17 in RT-based combination group, and 12 in RT only group had lesions only involved in original sites. Four patients in GKS-based combination group, 5 in RT-based combination group, and 3 in RT only group had tumors located in original and other sites. Twenty-two of these 70 patients with second CNS recurrent lesions received supportive care. The remaining 48 cases were treated with combination treatment strategies. Forty-two patients underwent GKS and chemotherapy and 6 received RT and chemotherapy.

**Table 5:** Location of second CNS recurrence after treatment.

	RT^a^ only (N = 16)	RT^a^ + other modalities^b^ (N = 29)	GKS only (N = 25)	GKS + other modalities^c^ (N = 20)
**No recurrence**	0 (0%)	7 (24.1%)	5 (20%)	5 (25%)
**Recurrence**				
Only original site	12 (75%)	17 (58.6%)	19 (76%)	10 (50%)
Original site + other sites	3 (18.75%)	5 (17.3)	0 (0%)	4 (20%)
**Not available**	1 (6.25%)	0 (0%)	1 (4%)	1 (5%)

CNS, central nervous system; +, and; RT, radiation therapy; GKS, gamma knife radiosurgery; N, number.

a RT included whole brain radiation therapy (WBRT) and spinal RT.

b Other modalities included chemotherapy, or surgery.

c Other modalities included RT, chemotherapy, or surgery.

### Potential of GKS-Based Treatment for EOC Patients With CNS Metastases

**Figure 4:**
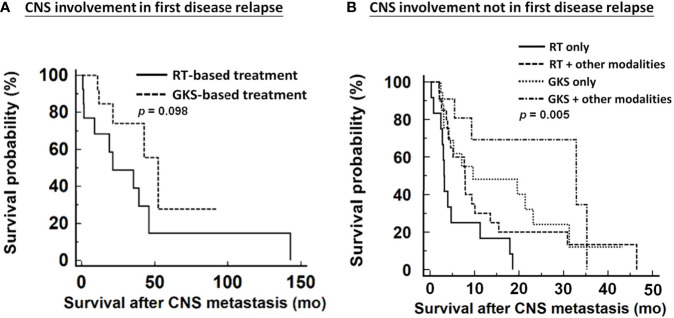
Impacts of various therapeutic modalities on survival outcomes of patients from diagnosis of CNS metastases. **(A)** patients with CNS involvement in first disease relapse, **(B)** patients with CNS involvement not in first disease relapse. CNS, central nervous system; RT, radiation therapy; GKS, gamma knife radiosurgery. RT included WBRT and spinal RT.

We further analyzed the impact of various therapeutic modalities on survival outcomes in patients diagnosed with CNS metastases at first tumor recurrence and those who were not. Because of the small number of EOC cases with CNS involvement in first disease relapse, these women could only be divided into two categories for survival analysis: GKS-based treatment and RT-based treatment. GKS-based treatment seemed to offer survival benefits over RT-based treatment without significance (median survival, 52.3 months *vs.* 21.3 months, respectively; *p*=0.098, **Figure 4A**). Among patients with CNS lesions not involved at first tumor recurrence, GKS-based combination regimens significantly demonstrated better survival than other treatment modalities (median survival, GKS-based combination group: 32.9 months; GKS only group: 9.7 months; RT-based combination group: 7.9 months; RT only group: 3.1 months; *p*=0.005, **Figure 4B**).

## Discussion

In this collaborative study, we investigated clinico-pathological risk factors and evaluated the potential therapeutic strategies for EOC patients with CNS metastases, including brain, spinal cord, and both. The incidence of CNS metastases in our cohort was 1.49%, which is similar to the results of previous studies on ovarian cancer patients with brain metastases (6, 12, 14). With the advancement of surgical techniques and availability of novel anticancer agents, the increasing number of patients with CNS metastases has been considered to be associated with improved survival of EOC as in the present study (12, 15, 16). However, compared to the incidence of CNS metastases from 2000 to 2009, a significantly increased incidence was not observed from 2010 to 2020 (1.31% vs. 1.61%). The reason may be the increasing incidence of ovarian cancer in recent years (17–19).

As reported in several surveys of ovarian cancer patients with brain metastases, most primary EOC patients in our studied population had serous histology, advanced disease status, and a good response to initial therapy (20, 21). At the diagnosis of CNS metastases, our analysis showed that most CNS tumors were not detected at first disease relapse and involved multiple CNS sites (6, 14, 15, 22). Although a third of patients were diagnosed as part of first-relapse episodes, we did not routinely examine the CNS during ovarian cancer surveillance because of low incidence. We usually performed the image study once the patients presented with any neurological disturbance. In this study, when CNS lesions were suspected, almost all patients (91/94, 96.8%) had at least one neurological disturbance. New-onset headache was the most common manifestation. However, in one of the largest series published thus far, approximately 26% of the studied population did not have symptoms at the diagnosis of brain metastases (15).

Several reports have demonstrated prognostic factors for brain metastases from ovarian cancer. Major factors that predict better survival include good performance status at diagnosis of CNS metastases, solitary intracranial lesion, absence of extracranial disease, absence of prior cancer relapse before brain metastases, platinum sensitivity, multimodal treatment, and application of GKS (6, 12, 14, 15, 20, 21, 23–27). In our study, serous histology, primary tumor without progressive disease after first-line treatment, CNS involvement in first disease relapse, and GKS-based therapeutic modalities were predictors of favorable survival outcomes in EOC with CNS metastases. For a gynecological oncologist, when CNS metastasis are identified, picking the optimal therapeutic treatment would be essential to improve survival.

As the time of occurrence of CNS relapse and treatment modalities for CNS metastases are two important factors affecting survival in this study, we performed a subgroup analysis. In order to investigate the impacts of different therapeutic strategies to treat patients with CNS involvement in first relapse or not, the effective modalities were evaluated in each clinical situations. This subgroup analysis showed superior survival in patients with CNS involvement not in first tumor recurrence who underwent GKS-based combination treatment compared to those who received other types of treatments. However, a survival benefit was not significantly noted in patients with CNS involvement in first disease relapse. However, a trend of longer survival was still observed in the GKS-based group among these patients.

In many institutions, conventional fractionated WBRT is still frequently applied as a standard therapy for CNS metastases. However, complications and limited local control with responses in 24-55% often cause unsatisfactory results (9, 28, 29), especially in larger brain metastases which may not be optimally controlled by traditional fraction sizes of ≤3Gy per day used in WBRT (10). Even without serious CNS toxicity in acute or early-delayed phase, delayed significant CNS toxicity of this fractionated radiotherapy still could be a concern (30). Recently, Butala et al. reported that cumulative biologically effective doses >39 Gy were associated with improved clinical response in CNS lesions without obvious toxicities, but there was a lack of information on tumor sizes in this study (31). GKS has overcome several of these limitations in a fundamental way. This modality is typically used for brain metastases ≤4 cm in maximum diameter, and prescription doses typically range from 15 Gy to 24 Gy for single-fraction sessions (10). It was associated with a high local control efficacy, avoidance of impaired cognitive function shorter hospital stay, less frequent and shorter steroid application, improved clinical performance, and lower frequency of toxicities (9, 10, 32, 33).

Currently, GKS is considered an alternative to surgical resection for small metastases without a mass effect in patients with tumors in or near the eloquent cortex, deep lesions, or high anesthetic risk (34). Although GKS provide well local control for the CNS lesions, patients who undergo GKS alone appear to have a higher incidence of local recurrence after treatment (35). Therefore, a rationale explaining the therapeutic effects of GKS-based combination regimens is that additional surgery, RT, or chemotherapy could control micro-metastases and reduce disease progression in CNS. In this study, the results of local tumor control were compatible with survival outcomes, which showed longest survival in patients treated with GKS-based combination treatment as initial treatment for metastatic CNS lesions. From this point of view, GKS-based combination treatment could provide effective treatment for CNS lesions, and relieve neurologic symptoms in a short period so that patients could receive systemic chemotherapy for extra-CNS tumors. Treatment for CNS metastasis from ovarian cancers has been remarkably changed over the past two decades. Most patients received RT (WBRT or spinal RT) 10-20 years ago. With the advancements and promising results of stereotactic radiosurgery for CNS tumor control, GKS has now become a more acceptable option to control CNS metastasis from ovarian cancers in recent 10 years. Although GKS-based combination treatment strategies have significant impacts on survival in EOC patients with CNS metastases, this phenomenon has not been observed in other types of cancer (36–40). The reasons for such differences are unclear but may be partially explained by differences in tumor biology and the use of small-molecule drugs that could cross the blood-brain barrier in other cancer types, especially breast and lung cancer (41, 42).

However, our retrospective study has some limitations. Because of the long timeframe for enrollment, we could not record detailed information on Karnofsky performance status (KPS) and evaluate the quality of life and cognitive or motor function before and after the treatment of CNS metastases, which should be an important issue for these patients. In addition, targeted therapy, such as bevacizumab and other small-molecule drugs, is currently used in EOC patients more extensively, but we still cannot elucidate their influence on CNS metastases from EOC.

In several reports, CNS metastasis from ovarian cancer was often considered to be late in the course of the disease with poor prognosis (7, 8, 14). However, we observed that one-third of patients in our study developed CNS metastatic tumors during the first episode of recurrence. A large population-based study also found 0.3% of patients with newly diagnosed ovarian cancers already had brain metastases (43). There was difference in survival among ovarian cancer patients with different CNS metastatic timing. In our study, patients with CNS metastases in first relapse had better survival outcomes compared with those with CNS metastases in late relapse, while patients with brain metastases at initial diagnosis of ovarian cancer had very poor outcomes with median survival of 2 months (43). Like our results, Xi et al. found that survivals of patients with brain metastases in newly diagnosed ovarian cancer can benefit from combined treatment (43). This interesting observation implies that there may be certain biological regulations behind tumor progression. Systemic chemotherapy seemed less likely to be the factor resulting in survival differences because it was the first therapeutic option to control extra-CNS in current clinical practice. Furthermore, none of patients in our cohort received intraperitoneal chemotherapy. Other factors influencing survival difference might be molecular alterations, including status of BRCA genes mutation. The detailed information was difficult to obtain in our study. Therefore, the regulatory mechanisms remain unknown which needs more efforts for investigation.

Although information of performance status was not readily available in this study, patients who received GKS-based treatment seemed to be with better performance status. Before starting GKS-based treatment, neurosurgeons evaluated these patients according to the requirements of GKS-based treatment. Thus, adoption of GKS can be a surrogate measure for patients’ performance status in this studied population. Our results showed patients who received GKS-base combination treatment had better survival outcomes, which may be partially caused from patients’ relatively good performance in addition to treatment itself. Apart from performance status, clinicians seemed to prefer adopting GKS-based combination treatment for patients with small tumor size, and small number of tumors. Patients with poor prognosis tended to receive palliative management. Those patients with disseminated extra-CNS lesions were also less likely to receive curative treatment like surgery or GKS-based treatment; therefore, survival outcomes of them were supposed to be worse. These selection bias were important concerns in our study due to its retrospective nature.

In conclusion, this is the first collaborative study to identify prognostic factors and potential therapeutic strategies for EOC with CNS metastases from two large academic institutions in Korea and Taiwan. Based on our analysis, GKS-based combination treatment modalities can be considered for the treatment of such cases. The patients with CNS involvement not in first disease relapse could significantly benefit from GKS-based combination strategies.

## Data Availability Statement

The raw data supporting the conclusions of this article will be made available by the authors, without undue reservation.

## Ethics Statement

The studies involving human participants were reviewed and approved by 1. Institutional Review Board of Samsung Medical Center 2. Institutional Review Board of National Taiwan University Hospital Hsin-Chu Branch. Written informed consent for participation was not required for this study in accordance with the national legislation and the institutional requirements.

## Author Contributions

Study conception and design: Y-LC, Y-YL. Acquisition of data: Y-LL, J-HK, and C-YH. Analysis and interpretation of data: all authors. Drafting of manuscript: Y-LL, J-HK, Y-LC, and Y-YL. Critical revision: all authors. All authors contributed to the article and approved the submitted version.

## Conflict of Interest

The authors declare that the research was conducted in the absence of any commercial or financial relationships that could be construed as a potential conflict of interest.

## Publisher’s Note

All claims expressed in this article are solely those of the authors and do not necessarily represent those of their affiliated organizations, or those of the publisher, the editors and the reviewers. Any product that may be evaluated in this article, or claim that may be made by its manufacturer, is not guaranteed or endorsed by the publisher.
